# The intratumoral administration of ferucarbotran conjugated with doxorubicin improved therapeutic effect by magnetic hyperthermia combined with pharmacotherapy in a hepatocellular carcinoma model

**DOI:** 10.1186/s13046-014-0057-x

**Published:** 2014-07-18

**Authors:** Min Jeong Jeon, Cheol-Hee Ahn, Hyeonjin Kim, In Jae Chung, Seulhee Jung, Young-Hwa Kim, Hyewon Youn, Jin Wook Chung, Young Il Kim

**Affiliations:** 1 Department of Radiology, Seoul National University College of Medicine, and the Institute of Radiation Medicine, SNUMRC, Seoul, Korea; 2 Department of Biomedical Sciences, Seoul National University College of Medicine, Seoul, Korea; 3 Department of Materials Science and Engineering, Research Institute of Advanced Materials, College of Engineering, Seoul National University, Seoul, Korea; 4 Department of Nuclear Medicine, Seoul National University College of Medicine, Seoul, Korea; 5 Laboratory of Molecular Imaging and Therapy, Cancer Research Institute, Seoul, Korea; 6 Department of Radiology, Seoul National University Hospital, 101 Daehak-ro, Chongno-gu 110-744, Seoul, Republic of Korea

**Keywords:** Hepatocellular carcinoma (HCC), Magnetic nanoparticle (MNP), Magnetic hyperthermia, Drug delivery system (DDS), Bioluminescence imaging (BLI)

## Abstract

**Background:**

Local hyperthermia of tumor in conjunction with chemotherapy is a promising strategy for cancer treatment. The aim of this study was to evaluate the efficacy of intratumoral delivery of clinically approved magnetic nanoparticles (MNPs) conjugated with doxorubicin to simultaneously induce magnetic hyperthermia and drug delivery in a hepatocellular carcinoma (HCC) model.

**Materials and methods:**

HCC cells expressing luciferase were implanted into the flank of BALB/c-nu mice (n = 19). When the tumor diameter reached 7–8 mm, the animals were divided into four groups according to the injected agents: group A (normal saline, n = 4), group B (doxorubicin, n = 5), group C (MNP, n = 5), and group D (MNP/doxorubicin complex, n = 5). Animals were exposed to an alternating magnetic field (AMF) to receive magnetic hyperthermia, and intratumoral temperature changes were measured.

Bioluminescence imagings (BLIs) were performed before treatment and at 3, 7, and 14 days after treatment to measure the tumoral activities. The relative signal intensity (RSI) of each tumor was calculated by dividing the BLI signal at each time point by the value measured before treatment. At day 14 post-treatment, all tumor tissues were harvested to assess the apoptosis rates by pathological examination.

**Results:**

The rise in temperature of the tumors was 1.88 ± 0.21°C in group A, 0.96 ± 1.05°C in B, 7.93 ± 1.99°C in C, and 8.95 ± 1.31°C in D. The RSI of the tumors at day 14 post-treatment was significantly lower in group D (0.31 ± 0.20) than in group A (2.23 ± 1.14), B (0.94 ± 0.47), and C (1.02 ± 0.21). The apoptosis rates of the tumors were 11.52 ± 3.10% in group A, 23.0 ± 7.68% in B, 25.4 ± 3.36% in C, and 39.0 ± 13.2% in D, respectively.

**Conclusion**s**:**

The intratumoral injection of ferucarbotran conjugated with doxorubicin shows an improved therapeutic effect compared with doxorubicin or ferucarbotran alone when the complex is injected into HCC tissues exposed to AMF for magnetic hyperthermia. This strategy of combining doxorubicin and MNP-induced magnetic hyperthermia exhibits a synergic effect on inhibiting tumor growth in an HCC model.

## Introduction

Hepatocellular carcinoma (HCC) remains the fifth most common cancer as well as the third leading cause of cancer mortality worldwide [[1]]. Current therapeutic options, including surgical resection, radiotherapy, and chemotherapy, have been unsatisfactory in most patients. Although surgical resection has been recognized the most effective treatment for HCC, its efficacy is limited to the minority of patients who have early stage disease. Patients with underlying liver disease, unsuitability for resection, or little organ availability for transplantation are not candidates for surgery [[2]].

Hyperthermia is a very promising cancer treatment based on the hypothesis that cancerous cells are more sensitive to an increase in the tissue temperature than normal cells [[3]]. In recent years, various hyperthermic ablation therapies such as radiofrequency ablation, microwave ablation, and high intensity focused ultrasound have been widely introduced especially for liver cancer. Another strategy for heat induction in tumor is magnetic hyperthermia. When exposed to a high-frequency magnetic field, magnetic nanoparticles (MNPs) generate heat through the oscillation of their magnetic moment due to Neel and Brownian relaxations [[4]]. Direct injection of MNPs into solid tumors, followed by exposure of tumors to an alternating magnetic field (AMF), has been shown to induce controlled heating at the target tumors, which leads to tumor regression [[5]]. After exposure of tumor-bearing organs to AMF, the induced heat that raises the tissue temperature to approximately 41–47°C is known to alter the function of many structural and enzymatic proteins within cells, which in turn arrests cell growth and differentiation and eventually induces apoptosis [[6],[7]]. This particle-induced magnetic heating can be controlled by accurate and localized delivery of the MNPs to the target lesions, and has been under several clinical trials [[8]]. Additionally, MNPs have been investigated as drug delivery systems to improve the efficacy of drugs. The loading of drugs to MNPs can be achieved either by conjugating the therapeutic agents onto the surface of the MNPs or by co-encapsulating the drug molecules along with MNPs within the coating material envelope [[9]]. Once at the target site, MNPs can stimulate drug uptake within cancer cells by locally providing high extracellular concentrations of the drug or by direct action on the permeability of cell membranes [[10]]. Most of MNPs are not approved for use in humans because their safety and toxicity have not been clearly documented. However, ferucarbotran (Resovist; Bayer Schering Pharma AG, Leverkusen, Germany) is a clinically-approved superparamagnetic iron oxide nanoparticle that has been developed for contrast-enhanced MRI of the liver [[11]]. Local hyperthermia of tumor tissue in conjunction with chemotherapy has been demonstrated to significantly enhance antitumor efficacy [[12]]. Here, we designed a complex made with both Resovist, an MNP approved for clinical use in humans, and doxorubicin to combine the magnetic control of heating and drug delivery into one treatment. We expected that this complex would enhance the synergistic efficacy and yield substantial promise for a highly efficient therapeutic strategy in HCC. The *in vivo* antitumor effect was evaluated by bioluminescence imaging (BLI), which measures the luciferase-expressing tumor cells’ activity, throughout the follow-up period.

## Materials and methods

### Preparation of the Resovist/doxorubicin complex

Doxorubicin was loaded on the surface of Resovist via an ionic interaction as previously described [[13]]. Resovist was loaded with doxorubicin through ionic interactions between anionically charged carboxydextran coating layer of Resovist and positively charged amino groups of doxorubicin. Predetermined amount of doxorubicin (0.2 mg, Adriamycin; Ildong Pharmaceutical, Seoul, Republic of Korea) was dissolved in 4 mL deionized water, and the aqueous solution was transferred to a 250-mL round-bottom flask. Diluted (1.38 Fe mg/mL) Resovist in 4 mL deionized water was added dropwise using a syringe pump at a rate of 0.1 mL/min, and the reaction mixture was vigorously stirred for 8 hours. Loading efficiency of doxorubicin was 100% and ultraviolet–visible spectroscopy at 480 nm confirmed that there was not any doxorubicin left in the aqueous solution. The Resovist/doxorubicin complex was obtained as a solid after freeze-drying and the diameter of the complex before and after the freeze-drying was not so different based on DLS data. The concentration of doxorubicin in the complex was adjusted to 1 mg/ml. The release profile of doxorubicin from the complex was evaluated by the dialysis method. Two milliliters aqueous solution of the complex conjugated to doxorubicin (2 mg) was transferred into a dialysis membrane with a molecular weight cutoff of 1 K and dialyzed against deionized water (20 mL). The temperature of the medium was changed to either 37°C or 60°C at a predetermined time, and an aliquot was sampled at 1, 2, 3, 4, 5, 6, 18, 42 and 66 hours. The amount of released doxorubicin was measured by ultraviolet–visible spectroscopy at 480 nm.

To test whether the conjugation process would affect the MR imaging of Resovist, we measured the MR relaxivity of the Resovist/doxorubicin complex, which was compared with that of Resovist. The particles were serially diluted from a concentration of 0.15 mM in an agarose phantom designed for relaxivity measurements, which was done using a 3-T MR scanner (Tim Trio; Siemens Healthcare, Erlangen, Germany). Fast spin echo T2-weighted MR images of the phantom were acquired using the following parameters: relaxation time = 5000 ms, echo times = 16, 32, 48, 64, 20, 40, 60, 80, 50, or 100 ms, flip angle = 180, ETL = 18 fields of view, FOV =77×110 mm^2^, matrix = 256×117, slice thickness/gap = 1.4 mm/1.8 mm, and NEX = 1.

### Preparation of the animal model

Hep3B, a human HCC cell-line, was transduced with a retroviral vector containing the firefly luciferase (luc) reporter gene, and a highly expressing reporter clone was isolated to establish Hep3B + luc cells. Hep3B + luc cells were cultured in Dulbecco’s modified Eagle’s medium (DMEM; Welgene, Seoul, Korea) supplemented with 10% (v/v) heat-inactivated fetal bovine serum (GIBCO, Seoul, Korea). All animal procedures were performed according to our Institutional Animal Care and Use Committee-approved protocol (SNUH-IACUC #12-0015). Male BALB/c-nude mice (n = 19), aged 6 weeks and weighing 20–25 g, were used for this study. Hep3B + luc cells were suspended at 1×10^6^ cells/0.1 ml in serum-free DMEM and subcutaneously injected into the right flanks of the animals. Two weeks after tumor implantation, when the tumor diameter reached approximately 7–8 mm in diameter, the animals were evenly divided into 4 groups according to the injected agents: group A (n = 4) injected with normal saline, group B (n = 5) with doxorubicin (4 mg/kg), group C (n = 5) with Resovist (Fe 111.6 mg/kg), and group D (n = 5) with the Resovist/doxorubicin complex (Fe 111.6 mg/kg, doxorubicin 4 mg/kg). As the lethal dose of ferucarbotran solution in rodents was reported to be in excess of 558 mg Fe/kg [[14]], our dosage of Resovist was within the safe range. All therapeutic agents were dissolved in the same volume of saline (0.1 ml) and injected directly into the core of tumors.

### Magnetic hyperthermia

**Figure 1:**
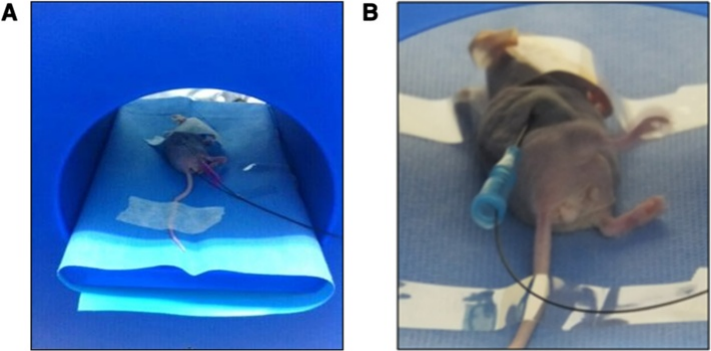
**Photograph of hyperthermia treatment. A)** A tumor-bearing mouse is placed in the center of the hyperthermia device generating AMF. **B)** A thermo-sensor is inserted into the tumor by way of a venipuncture catheter to measure temperature changes during the treatment.

The animals were fully anesthetized by intraperitoneal administration of 12 mg/kg tiletamine-zolazepam (Zoletil 50; Virbac, Carros, France) and 0.75 mg/kg xylazine hydrochloride (Rompun; Bayer, Seoul, South Korea). The animals were then placed in the center of AC coil to generate AMF (Figure 1). An original device was connected to the coil (width 30 cm, length 30 cm) and cooling unit, which was cooled continuously by flowing water by the unit (Recirculating coolers HX-45H; Jeiotech, Daejeon-si, Korea). A high-frequency generator worked at a current of 155 Oe at a frequency of 100 kHz for magnetic hyperthermia. A 20-gauge venipuncture catheter (BD Angiocath Plus with intravenous catheter; Becton Dickinson Korea, Gumi-si, Korea) was inserted into each tumor so that an electronic thermometer (Luxtron m3300 Biomedical Lab Kit Fluoroptic Thermometer; LumaSense Technologies, Santa Clara, CA) could be passed through the catheter to measure the core temperature of the tumor during the procedure. To evaluate the selectivity of heating during the hyperthermia treatment, rectal temperatures were simultaneously measured in a same manner as described above.

### Bioluminescence imaging for the *in vivo* evaluation of therapeutic responses

Bioluminescence imaging (BLI) was performed using the IVIS lumina II (PerkinElmer, Waltham, MA). Mice were anesthetized with 1% isoflurane (Ifran, Hana Pharm. Co, Seoul, Korea) in room air. D-luciferin (Caliper Life Sciences, Hopkinton, MA) dissolved in PBS (1.5 mg luciferin/100ul PBS) was injected intraperitoneally at a dose of 150 mg luciferin/kg, and serial images were acquired with an exposure time of 30 sec, an f/stop of 1, and pixel binning at 8 over 20 minutes to determine the peak bioluminescence. Subsequently, regions of interest (ROIs) of equal size were drawn within the tumor to measure average radiance (expressed as photons/s/cm2/sr). The BLIs were performed just prior to treatment to obtain the baseline value and at 3, 7 and 14 days after treatment. By using Living Image® 4.2 software (Caliper Life Sciences, Hopkinton, MA), we measured the peak total tumor bioluminescent signal through standardized ROIs. To ensure longitudinal comparability of the serial measurements, we calculated the relative signal intensities (RSIs) by normalizing each measured peak total tumor bioluminescent signal in a mouse with the signal at baseline as follows: [RSI at a time-point = (peak signal intensity at a time-point/peak signal intensity at baseline)] [[15]].

### Histopathological evaluations

All animals were euthanized at day 14 after treatment. The extracted tumors were perfused with PBS, fixed in 4% paraformaldehyde solution, and embedded in paraffin. The tumors were sectioned at a thickness of 4 μm at the largest tumor area. Hematoxylin and eosin (H&E) staining was performed for a general inspection of the pathologic specimens. Prussian blue staining was added to visualize the injected iron particle distribution within the tumor tissues. To evaluate the extent of tumor apoptosis for validating *in vivo* BLI results, a terminal deoxynucleotidyl transferase dUTP nick end labeling (TUNEL) assay was performed with a commercial kit (Roche, Mannheim, Germany). TUNEL staining is a method to stain cells exhibiting apoptotic or non-apoptotic DNA damage (i.e., DNA fragmentation), such as necrotic cell death [[4],[16]]. The percent area of apoptosis was calculated using NIH Image J software (NIH, Bethesda, MD). After drawing a free-hand ROI to completely cover the tumor, the number of pixels in the tumor area was counted. Within the selected tumor area, the number of pixels corresponding to the apoptosis area stained with TUNEL was also counted. The percent area of apoptosis (%) was calculated by dividing the area of the TUNEL-stained area (pixels) by the area of the total tumor (pixels).

### Doxorubicin fluorescence microscopy

Fourteen days after treatment, some of the extracted tumor tissues were immediately cryosectioned at a thickness of 6 μm in the largest tumor and stored at −70°C. After washing the tissues, the cover slips were mounted onto glass slides using mounting medium (Faramount aqueous mounting medium; Dako, Carpinteria, CA). On the slides, the distribution of doxorubicin over the tumor area was observed under a fluorescence microscope (Leica DM5500B, Leica, Wetzlar, Germany) using excitation and emission wavelengths of 520 and 580 nm, respectively. The fluorescence images were acquired using the following parameters: magnification = 200×, BF: EX14 Gain 1.1 Intensity 1 gamma 45, and FLU: EX 656 Gain 4 Intensity 5 gamma 20.

### Statistical analyses

All data are expressed as means ± standard deviation (SD), and the data processing and analysis were performed using SPSS version 16.0 (SPSS, Inc., an IBM Company, Chicago, IL). The nonparametric analysis was conducted by the Mann–Whitney test to compare the temperature changes in tumors, BLIs values, and apoptosis rates between the experimental groups. A *p*-value of less than 0.05 was considered statistically significant.

## Results

### The characterization of the Resovist/doxorubicin complex

**Figure 2:**
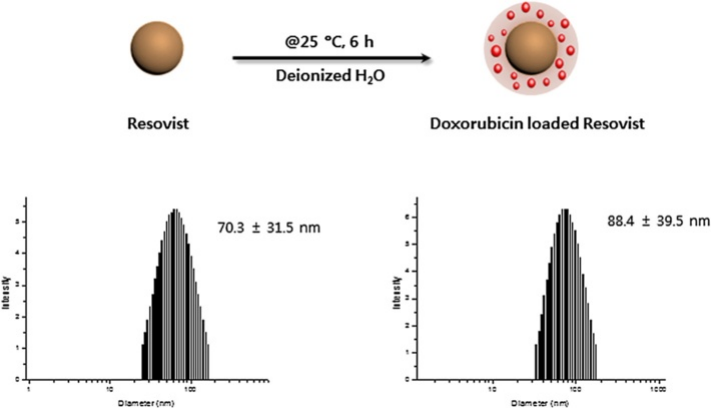
Preparation and characterization of Resovist-doxorubicin complex.

**Figure 3:**
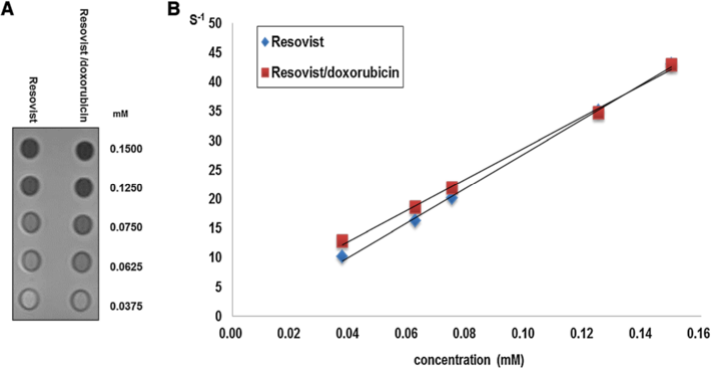
**Measurement of MR relaxivities. A)** T2-weighted MR image of the phantom for relaxivity measurement. **B)** Plot of the inverse transverse relaxation times (1/T2) vs. Fe concentration. The slopes indicate the specific relaxivity value (r2).

The size of Resovist measured by dynamic light scattering was 70.3 ± 31.5 nm and increased to 88.4 ± 39.5 nm when doxorubicin was conjugated on the surface (Figure 2). The amount of doxorubicin was adjusted to be 2 mg/mL at the final administration for our study. Because the amount of doxorubicin was not a maximized value, the loading efficiency was 100%. The Resovist/doxorubicin complex was freeze-dried and stored as a solid. Redispersion of the complex by vortexing and/or sonication resulted in a similar size distribution reproducibly without any difficulties. When measuring the T2 relaxivities of the particles, the r_2_ values of Resovist and the Resovist/doxorubicin complex were 295.0 s^−1^ mM^−1^ and 265.7 s^−1^ mM^−1^, respectively (Figure 3).

**Figure 4:**
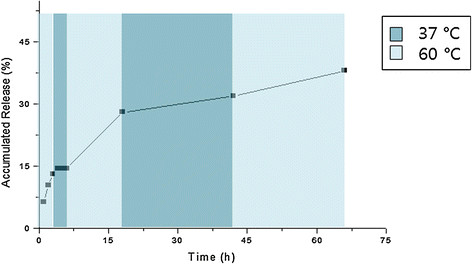
**The** ***in vitro*** **release pattern of doxorubicin from the Resovist-doxorubicin complex.**

Figure 4 summarizes the release pattern of doxorubicin from the complex. The driving force for the doxorubicin conjugation is an ionic interaction, which is known to weaken as the temperature increases. The release test was performed at two different temperature, 37°C and 60°C, with a predetermined time profile to mimic the condition of hyperthermal therapy. As expected, sustained release of doxorubicin was observed at 37°C, whereas the release was accelerated at the elevated temperature.

### Tumor temperature measurement

**Figure 5:**
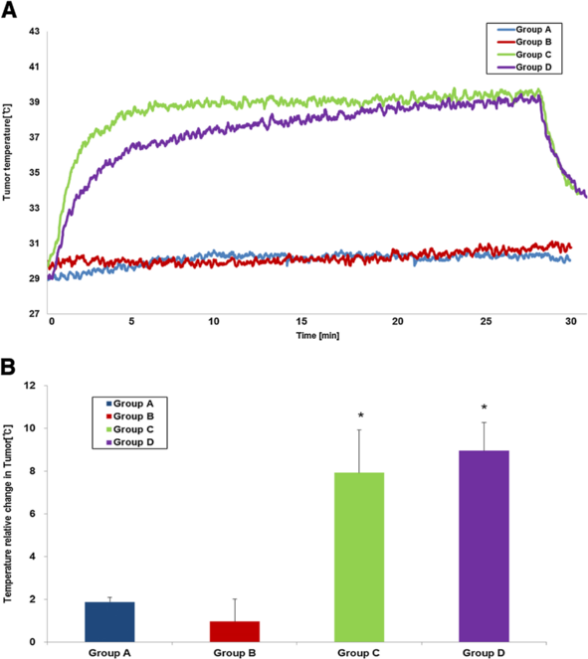
**The temperature changes of the tumors. A)** Plot of the temperature change curve during heating versus time (blue: group A, red: group B, green: group C, purple: group D). **B)** The mean temperature changes of the tumors (t/t0) during treatment. The error bars represent the standard deviations (*P < 0.05, compared to group A).

The tumor temperature in group C and D rapidly increased to approximately 42°C within 5 minutes and then remained stable for 20 minutes, whereas in group A and B did not increased significantly (Figure 5A). The average values of tumor temperature change 25 minutes after initiation of hyperthermia were 1.88 ± 0.21°C in group A, 0.96 ± 1.05°C in group B, 7.93 ± 1.99°C in group C, and 8.95 ± 1.31°C in group D (Figure 5B). Group C and D exhibited a significantly higher temperature in the tumors than group A or B (*p* < 0.05). The exact *p*-values obtained from comparisons between groups are summarized in Table 1. The rectal temperatures in all groups remained stable near the baseline values during the treatment.

**Table 1:** Comparisons of the temperature changes in tumor, RSIs of BLI at day 14 post-treatment, and apoptosis rates between groups (*p < 0.01, **p **< 0.05)**

	**Group B vs. C**	**Group B vs. D**	**Group C vs. D**
**Temperature changes**	0.009*	0.009*	0.465
**RSIs of BLI**	0.834	0.047**	0.009*
**Apoptosis rates**	0.675	0.028**	0.008*

Each number in the table indicates a *p*-value obtained by Mann–Whitney test.

### Bioluminescence imaging findings

**Figure 6:**
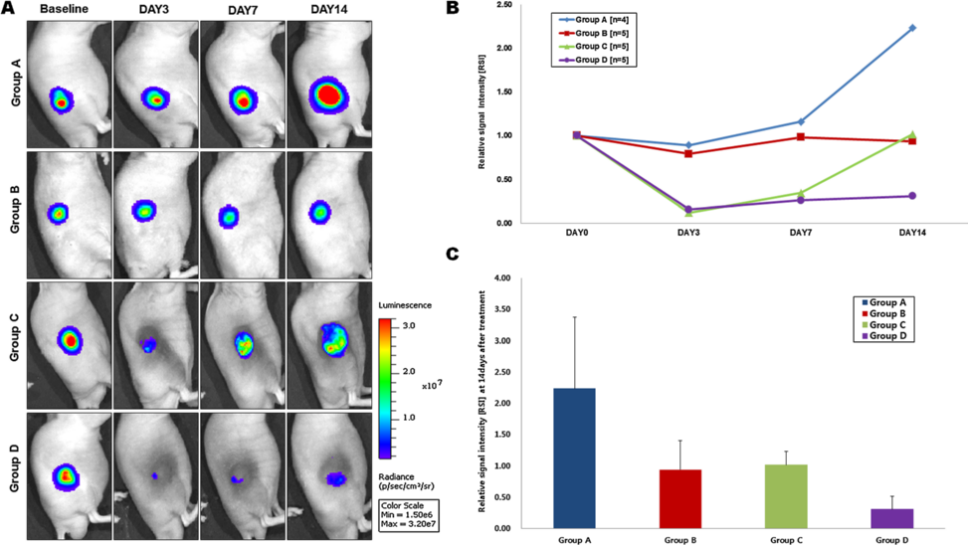
**Bioluminescence imaging (BLI). A)** Representative BLI obtained in each group by the IVIS lumina II (PerkinElmer, Waltham, MA). **B)** Relative signal intensity [RSI] of BLI over the follow-up period. **C)** A graph demonstrated the relative signal intensity [RSI] of BLI at 14 days after treatment (*P < 0.05, compared to group A).

In group A receiving normal saline for control, the RSI of BLI increased continuously over the follow-up period reflecting active tumor growth (2.23 ± 1.14). In group B, the RSI of BLI slightly decreased gradually until day 14 post-treatment (0.94 ± 0.47), which suggests that the cytotoxic effect of doxorubicin works on the tumor slowly (Figure 6A, B).In group C, the RSI of the BLI rapidly dropped 3 days after treatment and rebounded to near-baseline value at day 14 post-treatment(1.02 ± 0.21), suggesting complete recovery of tumoral activities at the later stage of treatment (Figure 6A, B). However, the RSI of BLI in group D dropped 3 days after treatment as in group C and exhibited minimal recovery until day 14 post-treatment (0.31 ± 0.20) (Figure 6A, B).The Mann–Whitney test performed for the BLI values at day 14 post-treatment revealed that the RSI of BLI in group D was significantly lower than the other groups (all *p*-values < 0.05) (Figure 6C). The exact *p*-values obtained between groups are summarized in Table 1. No mouse exhibited signs of debilitation in any of the groups through the follow-up period.

### Histopathological findings

**Figure 7:**
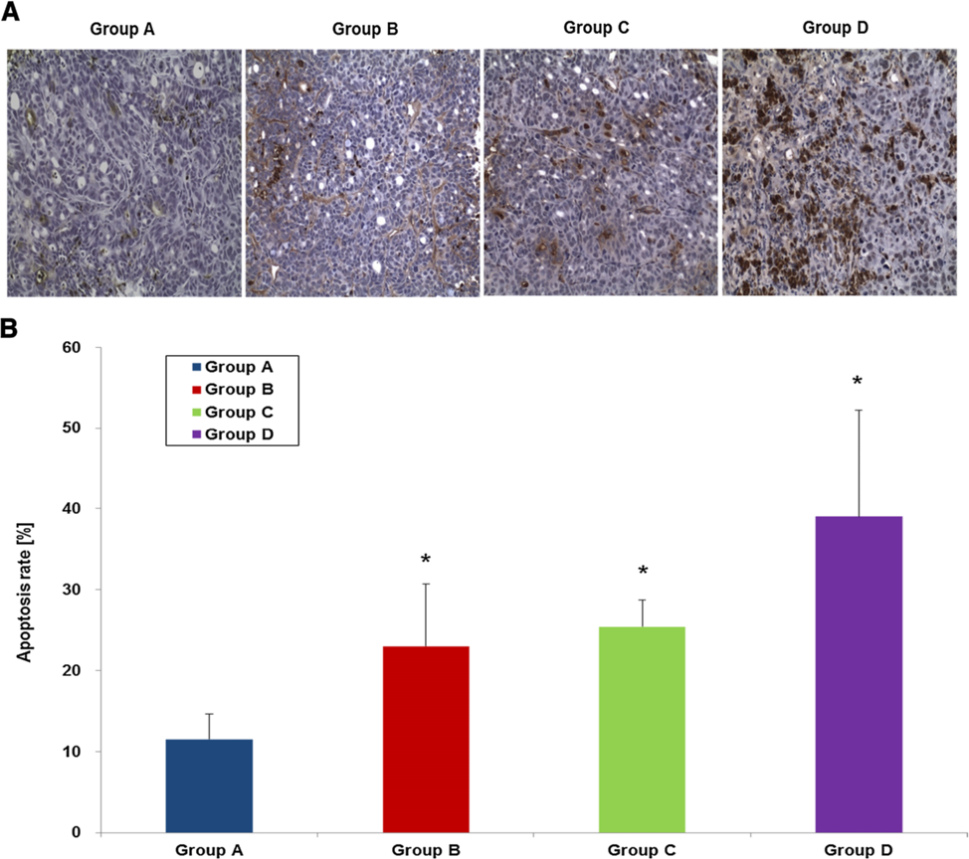
**Terminal deoxynucleotidyl transferase-mediated nick end labeling (TUNEL) assays to measure apoptotic cell death by light microscopy. A)** TUNEL-positive (brown color) cells with apoptotic morphology were observed in all groups (x200). **B)** A graph demonstrating the apoptosis/necrosis rates in all groups by image J software (*P < 0.05, compared to group A).

**Figure 8:**
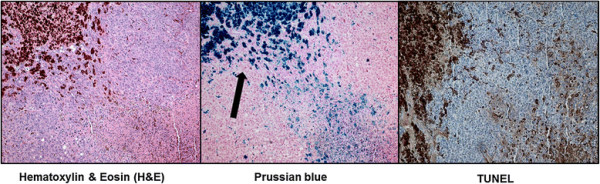
**Histopathological analyses of the tumor tissues by light microscopy.** Hematoxylin and eosin staining (left), Prussian blue staining (middle), and TUNEL staining (right) of a tumor treated with the Resovist/doxorubicin complex (x100).

TUNEL assay of the tumor tissues obtained at day 14 by revealed that the apoptosis/necrosis rate in group D was higher (39.0 ± 13.2%) than group A (11.52 ± 3.10%), B (25.4 ± 3.36%), and C (23.0 ± 7.68%) (Figure 7). Therefore, the Resovist/doxorubicin complex showed significantly more cell death than doxorubicin or Resovist monotherapy (all *p*-values < 0.05).The exact *p*-values obtained between groups are summarized in Table 1. Prussian blue staining of the consecutive section demonstrated multiple iron deposits within the tumor tissues in groups C and D (Figure 8).

### Doxorubicin fluorescence microscopic findings

**Figure 9:**
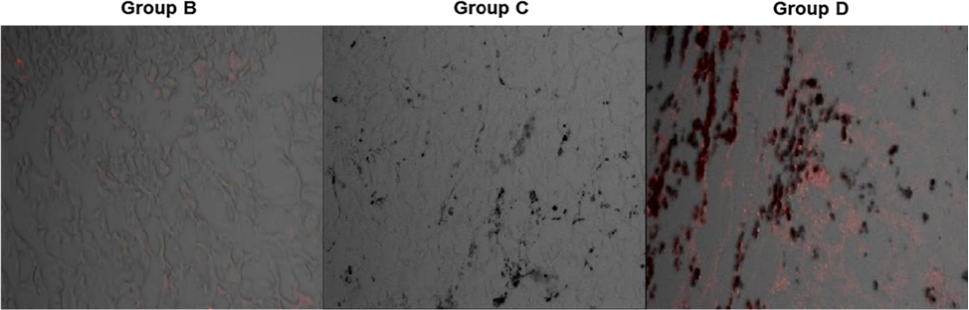
**Fluorescence microscopy images.** Representative fluorescence images of **group B** (left), **group C** (middle), and **group D** (right). **Group D** exhibits higher doxorubicin fluorescence signals (red spots) than the other two groups.

On fluorescence microscopic examination, group D exhibited higher fluorescence intensity from doxorubicin in the tumor tissues, which significantly overlapped the area with the iron particles. By contrast, group B exhibited minimal fluorescence from doxorubicin (Figure 9). This result suggests that the Resovist/doxorubicin complex could release doxorubicin into the tumor tissues in a controlled manner for a longer period than free doxorubicin and allowed persistent drug accumulation.

## Discussion

MNPs have gained considerable interest for biomedical applications over the past two decades [[17]]. Although this excitement has been driven mostly by the success of MNPs as T2 MR contrast agents [[18]], the recent investigative trend has turned toward therapy with respect to cancer. The key properties of MNPs for cancer include drug delivery, magnetic hyperthermia, and MR imaging. Thus, MNPs contribute both diagnostic and therapeutic accomplishments in a single system.

Drug delivery systems are required to ensure that the drug is properly delivered to target, and nanoparticle-based drug delivery systems have been developed as potential drug carriers for decades. Because the large surface-to-volume ratio of MNPs, like other nano-carriers, enables a high loading of various functional ligands on a single platform, marked attention has been paid to their use as drug delivery vehicles. In our study, the loading efficiency of doxorubicin was 100%. The ultraviolet–visible spectroscopy at 480 nm confirmed that there was not any doxorubicin left in the aqueous solution, which led to a conclusion that washing step to remove unbound doxorubicin was not required.

MNP coatings provide anchor points to which drug molecules can be coupled and have incorporated traditional small molecules such as doxorubicin for cancer therapy [[19]], as in our study. Resovist is coated with carboxydextran, to which doxorubicin was linked via ionic complexation by dropping synthesis with an average size of less than 100 nm in our study (Figure 2). When Resovist/doxorubicin complex reached tumor tissues after intratumoral injection, the complex was able to carry higher concentrations and exhibited prolonged release of doxorubicin in the tumor tissues as measured by fluorescence microscopy (Figure 9).

Magnetic hyperthermia can be used to selectively kill tumor cells via increases in tissue temperature [[4]]. When MNPs accumulating at the tumor site are exposed to AMF, MNPs absorb this energy and convert it into heat owing to the relaxation of the rotating magnetic moments induced by the AC field. Tumors are usually heated to the temperature range of 41–47°C, and cancer tissues exhibit higher heat sensitivity than normal tissues [[20]]. It also has been believed that the drug delivery to target could be increased by hyperthermia through its effects on convection and diffusion in tissues, increasing cell uptake of the drug, tumor blood flow and vascular permeability [[21]]. In our study, Resovist or the Resovist/doxorubicin complex also induced temperature increases to approximately 41°C (Figure 5A). Although magnetic hyperthermia is a promising cancer therapy, the risk of local overheating (and thus damage to normal tissues) remains the major concern, as in other clinical hyperthermia therapies such as radiofrequency ablation or high-intensity focused ultrasound. To overcome these challenges, the MNPs should be accurately delivered only to the target tumors, the temperature of which can be easily controlled by adjusting the MNP concentration delivered and the proper manipulation of the magnetic field strength. Furthermore, some thermally responsive agents that aid in specific nanoparticle retention within the tumor can reduce the diffusion of MNPs to healthy tissues adjacent to the tumor [[22]]. One of the advantages of magnetic hyperthermia over other clinical hyperthermic treatments is that one is able to repeat the treatment in a short interval without additional invasive procedures. MR scans can predict the distribution of the MNPs to prevent unwanted heating of the normal tissues. If the nanoparticles accurately cover the tumor tissues on a short-term follow-up MR, magnetic hyperthermia is able to be repeated without causing major side effects. Furthermore, local overheating may be avoided by selecting particles with a low maximal achievable temperature while preserving the magnetization for efficient heating [[23]]. Among the many MNPs, Resovist is clinically approved for contrast-enhanced MR in human [[11]] and was previously reported to generate effective heat in AMF [[14]]. Choosing an MNP already approved for clinical use was our main strategy to facilitate early translation of our study into clinical practice. Though Resovist is not marketed as a MR contrast agent due to the emergence of a novel MR contrast, the result in our study may open a new potential other than MR contrast for its clinical use.

Ferucarbotran consists mainly of a hydrophilic colloidal solution of superparamagnetic iron oxide coated with carboxydextran. It is a complex composed of ultrafine (7nmdiameter) magnetite particles and alkali-treated dextran [[4]]. The tumor cells in the center of the tumor tissues are not sensitive to chemotherapy due to hypoxia but are sensitive to hyperthermia due to low pH value, whereas the tumor cells in the tumor periphery are sensitive to chemotherapy [[12],[24]]. Hyperthermia, when it is applied to specific lesions, produces increased perfusion to the diseased area and makes the cells more permeable for better cellular uptake of agents. Therefore, when the hyperthermia is combined with chemotherapy for cancer, the heat that is generated in the targeted tumor can induce higher levels of drug accumulation in the tumor cells by the same mechanism described above. Doxorubicin is visualized by fluorescence microscopy with excitation wavelength at 480 nm [[25]], which enables us to detect the doxorubicin deposits in the tumor tissues. In our study, the fluorescence intensity was much higher in group D than in group B, suggesting an increased and long-lasting uptake of doxorubicin into the cells in group D (Figure 9).

Although doxorubicin has been widely used as single agent or in combination with other anticancer drugs for HCC [[26]], the drug produces many side effects derived from its nonspecific uptake into healthy normal tissues [[27]]. Therefore, recent studies have focused on the development of administration routes for doxorubicin to increase tissue selectivity [[28]]. Local administration of the agent is one promising approach with the advantage of reaching high concentrations at the target site more effectively than systemic delivery [[29]]. Although we injected the therapeutic agents directly into the tumor by the naked eye in our study, we are designing a future project to create an orthotopic liver tumor in which we can inject the therapeutic agents under image guidance using ultrasonography. Our future experiment using an orthotopic model is expected to provide more translatable data.

In this study, we performed BLI for *in vivo* monitoring of the therapeutic effect. BLI requires a reporter construct produce luciferase, an enzyme that provides imaging contrast by light emission resulting from luciferase-catalyzed conversion of D-luciferin to oxyluciferin in small animals [[30]]. Our data demonstrated that the tumor activity signals in group D were significantly lower than those in groups B and C at the end of follow-up period (Figure 6). Fourteen days after treatment, the BLI signal intensity reverted to 31% of the baseline value in group D, whereas those of groups B and C reverted to 90% and 113%, respectively. Although hyperthermia applied in the absence of doxorubicin exhibited a marked reduction in the BLI signal in the early stages of treatment, the signal was fully recovered at day 14 post-treatment. However, combination therapy using the Resovist/doxorubicin complex demonstrated a BLI signal that did not rebound during the 14 days post-treatment, representing persistent antitumor efficacy.

In conclusion, the biomedical application of nanomaterials is gradually increasing and is a challenging area for future research. Despite the a significant progress with respect to MNP platforms, regulatory approval for use in humans requires extensive safety studies of newly developed particles. To overcome challenges for clinical translation, we proposed an innovative approach that exploits MNPs conjugated with an anti-cancer drug to achieve efficient drug release and thermotherapy in a single platform composed of agents already approved for use in humans. We determined that combination therapy using the Resovist/doxorubicin complex could enhance anti-tumor efficacy in an HCC model by simultaneous induction of hyperthermia and drug delivery. This system enables a multi-modal therapy that can provide an efficient strategy against cancer based on both physical (heat) and chemical (drug) properties. We hope that our results will help to facilitate the clinical translation of MNPs for their future development.

## Competing interests

The authors declare that they have no competing interests.

## Authors’ contributions

MJJ participated in study design, in vivo studies, data analysis, and manuscript drafting. CHA participated in study design, in vitro studies, data analysis, and manuscript drafting. HK and JWC participated in study design, and interpretation of data. IJC and SJ participated in in vitro studies, and data analysis. YHK and HY participated in in vivo studies, and data acquisition. YlK participated in study design, in vivo studies, data analysis, and manuscript drafting, and critical revision of the manuscript. All authors read and approved the final manuscript.

## Funding

This work was supported in part by the Basic Science Research Program through the National Research Foundation of Korea funded by the Ministry of Education, Science and Technology (2011–0010250), and the Korean Health Technology R&D Project, Ministry of Health & Welfare, Republic of Korea (HI12C1148).
